# Percutaneous transhepatic papilla balloon dilatation combined with a percutaneous transcystic approach for removing concurrent gallbladder stone and common bile duct stone in a patient with billroth II gastrectomy and acute cholecystitis: A case report

**DOI:** 10.1097/MD.0000000000007964

**Published:** 2017-09-01

**Authors:** Dong Li, Yu-Liang Li, Wu-Jie Wang, Bin Liu, Hai-Yang Chang, Wei Wang, Yong-Zheng Wang, Zheng Li

**Affiliations:** a Department of Interventional Medicine, The Second Hospital of Shandong University; b Intervention Research Institute of Shandong University, Jinan, China.

**Keywords:** acute cholecystitis, common bile duct stone, gallbladder stone, percutaneous transcystic, percutaneous transhepatic papilla dilation

## Abstract

**Background:**

A 61-year-old man presented with upper abdominal pain and jaundice. Abdominal computed tomography imaging revealed stones in the gallbladder and the common bile duct, with a thickening of the gallbladder wall and an obvious increase in the volume of the gallbladder. Initial treatment using endoscopic retrograde cholangiopancreatography failed due to the presence of surgically altered gastrointestinal anatomy. Stones in the gallbladder and common bile duct were subsequently removed concurrently via percutaneous transhepatic papilla balloon dilatation combined with a percutaneous transcystic approach. Liver function recovered rapidly, with total bilirubin and direct bilirubin levels decreasing to normal, with a concomitant improvement in hemoglobin and thrombocyte levels and resolution of the upper abdominal pain and jaundice.

**Conclusion:**

Percutaneous transhepatic papilla balloon dilatation, combined with a percutaneous transcystic approach, provided an effective alternative treatment for removing concurrent stones in the common bile duct and gallbladder in a patient with a previous Billroth II gastrectomy and presenting with an acute cholecystitis.

## 1 Introduction

Biliary lithiasis is a worldwide disease that affects 20% of the general population, with approximately 15% of patients presenting with concurrent gallbladder (GB) and common bile duct (CBD) stones.^[1,2]^ Laparoscopic cholecystectomy (LC) is regarded as the standard treatment for GB stones, with endoscopic stone extraction combined with LC widely accepted as the treatment of choice for the treatment of concurrent GB and CBD stones.^[3]^ However, transpapillary endoscopic approaches, such as endoscopic retrograde cholangiopancreatography (ERCP) and endoscopic sphincterotomy (EST), are technically challenging to perform in patients with surgically altered gastrointestinal anatomy, and Roux-en-Y reconstruction in particular, due to difficulty in cannulating the bile duct.^[4]^

In the patient, we present in this case report, ERCP had previously been attempted but failed because of the presence of surgically altered gastrointestinal anatomy. Consequently, we performed percutaneous transhepatic papilla balloon dilatation (PTBD) combined with a percutaneous transcystic approach as a 1-step procedure for removal of concurrent GB and CBD stones. The consent for case publication was obtained from the patient.

## 2 Case report

**Figure 1:**
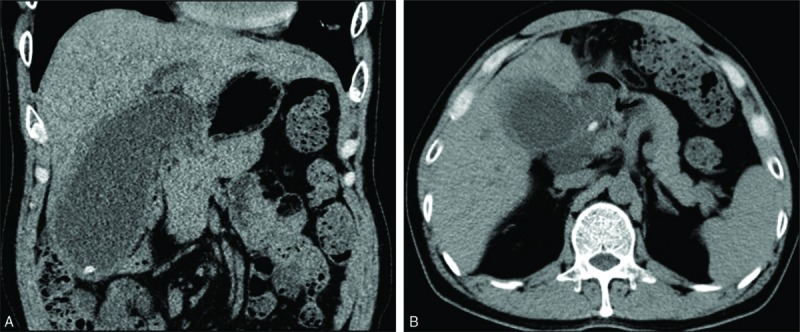
Computed tomography (CT) image showing (A) the gallbladder stone and (B) the combine bile duct stone. CT = computed tomography.

A 61-year-old man presented to our hospital with upper abdominal pain and jaundice. Abdominal computed tomography imaging revealed a 5-mm stone in the inferior portion of the gallbladder (Fig. 1A) and an 8 mm CBD stone (Fig. 1B). A thickened gallbladder wall and an obvious increase in the volume of the gallbladder were also observed. The patient had previously undergone a Billroth II surgery for treatment of a gastric carcinoma, 12 years earlier. Surgical treatment had been followed by FOLFOX4 combination chemotherapy, consisting of oxaliplatin 85 mg/(m^2^•d), ivgtt, d_1_, leucovorin 200 mg/m^2^, ivgtt 2 hours followed by 5-fluorouracil 400 mg/m^2^, and 5-fluorouracil 600 mg/m^2^ (22 h-continuous infusion). The FOLFOX4 regimen was repeated at a 2-week interval, with 4 weeks to complete 1 cycle.

For the treatment of GB and CBD stones, the patient refused treatment using an open or laparoscopic approach because of the presence of abdominal lymph node metastases. As such, ERCP was selected as the first treatment approach. ERCP was performed by one of the specialists in our digestive system department at our hospital but failed because of the previous Billroth II procedure. Consequently, we proceeded with PTBD combined with a percutaneous transcystic approach for concurrent removal of the GS and CDB stones. Written informed consent was obtained from the patient, preoperatively.

**Figure 2:**
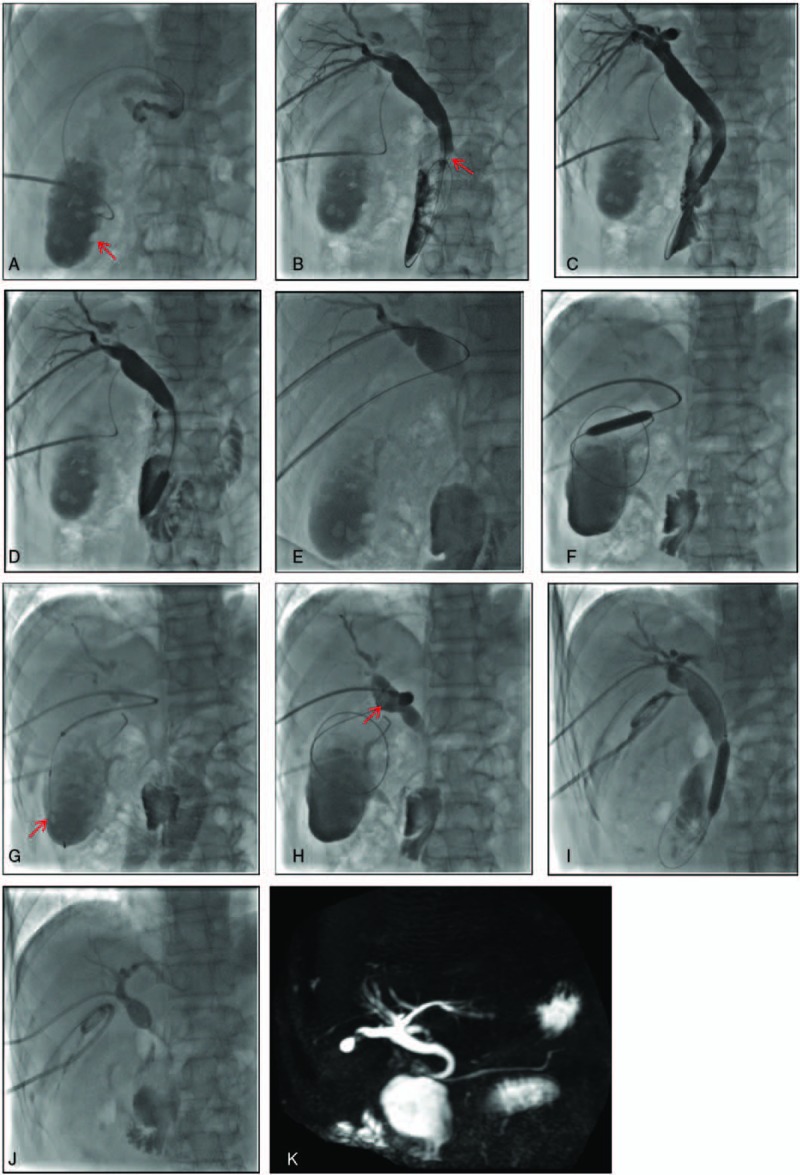
(A) Percutaneous transcystic cholangiography, showing the gallbladder stones and the guide wire, passed through the cystic duct and into the common bile duct. (B) Percutaneous transhepatic cholangiography, showing the common bile duct stone and guide wire, passed through the papilla and into the duodenum. (C) The papillary was dilated using a 40 × 10 mm balloon. (D) The common bile duct stone was pushed out into the duodenum by an inflated balloon. (E) A clear approach through the gallbladder, cystic duct, common bile duct, and intrahepatic bile duct was established. (F) The cystic duct was dilated using a 40 × 8 mm balloon. (G) A stone basket was place into the gallbladder. (H) The gallbladder stone was removed to the common bile duct and (I) pushed into the duodenum using the dilated balloon. (J) Two drainage catheters were placed in the common bile duct and the gallbladder. (K) A MRCP was performed to identify that there were no residual stones in GB and CBD.

Routine laboratory blood tests, electrocardiography, and evaluation of hepatic and renal function were completed, preoperatively. The patient completed a 4 hour fast prior to surgery. Following conscious sedation, surgery was performed under Doppler ultrasound guidance and fluoroscopic monitoring. A 21-gauge needle (Cook Medical, Bloomington) was first used to puncture the gallbladder and percutaneous cholangiography was performed through a 6-French introducer sheath (Terumo, Tokyo, Japan). Multiple positive and negative GB stones were identified. Next, a short guidewire (diameter, 0.035 inch [0.88 mm]); Terumo, Tokyo, Japan) was placed into the CBD via the cystic duct (Fig. 2A). The right hepatic duct was then punctured with a 21-gauge needle, and the biliary system was visualized by cholangiography. A short guidewire (diameter, 0.035 inch [0.88 mm]) was then inserted into the CBD through a 6-French introducer sheath through the transhepatic route. A second guidewire (length, 260 cm; diameter, 0.035 inch [0.88 mm]) was passed through the papilla and into the duodenum (Fig. 2B). A 40 × 10 mm balloon (Balt Extrusion, Montermorency, France) was advanced through the guidewire and into the duodenum. Under fluoroscopic guidance, the middle part of the balloon was placed at the sphincter (Fig. 2C), and the balloon was slowly inflated with a contrast agent to a maximum pressure of 6 to 8 atmospheres. The maximum inflation was retained for 10 to 20 s, until the waist of the balloon could no longer be seen. The balloon was partially deflated and pulled to the CBD stone and used to push the stone into the duodenum (Fig. 2D). A snare (Merit Medical, South Jordan) was then advanced through the transhepatic route to the CBD to catch and retrieve the guidewire delivered from the transcystic route, and a clear approach through the gallbladder, cystic duct, CBD, and intrahepatic bile duct was established (Fig. 2E). The cystic duct was dilated using a 40 × 18 mm balloon (Balt Extrusion, Montermorency, France) and a stone basket (Olympus, Tokyo, Japan) was advanced through the transhepatic route into the gallbladder to remove the GB stones into the CBD (Fig. 2F–H). The GB stones were pushed into the duodenum through the dilated sphincter of Oddi one by one, using the inflated balloon (Fig. 2I). Intraoperative cholangiography was performed to ensure the absence of any residual stones. Finally, two 8.5 French external drainage catheters (Cook Medical, Bloomington) were placed in the CBD and the gallbladder (Fig. 2J). On postoperative day 6, a repeat cholangiography was performed to confirm the absence of any residual stone and patency of the CBD, and the drainage catheter in the CBD was removed. The drainage catheter in the gallbladder was removed at 1-month postoperatively. A MRCP (magnetic resonance cholangiopancreatography) was performed to identify that there were no residual stones in GB and CBD (Fig. 2K). The liver function test and levels of hemoglobin and thrombocyte were monitored until the patient was fully recovered. Liver function recovered rapidly, with total bilirubin and direct bilirubin levels decreasing to normal. As well, hemoglobin and thrombocyte levels improved (Table 1), with resolution of the upper abdominal pain and jaundice.

**Table 1:**
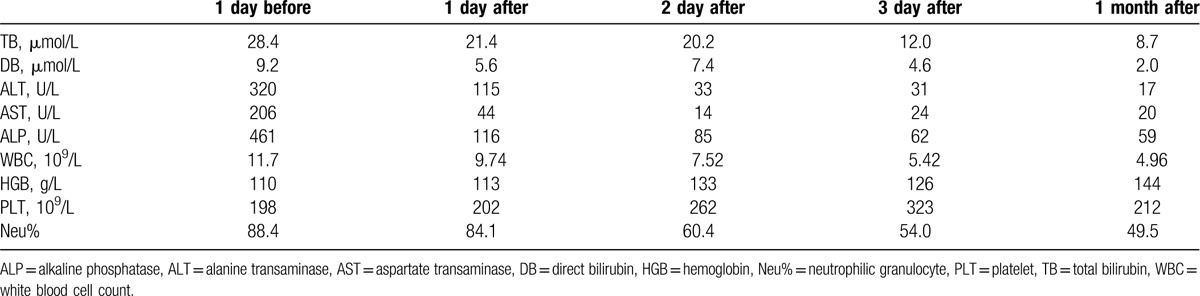
Patient's laboratory findings before and after the procedure.

## 3 Discussion

Approximately 15% of patients with GB stones present with concurrent CBD stones.^[2]^ Endoscopic stone removal is the preferred and most common approach for CBD stone removal in most countries.^[5]^ Since 1974, EST has been considered as the main treatment for CBD stones in most countries.^[4]^ For patients with concurrent GB stones, subsequent LC is considered as routine treatment.^[6]^ In our case, the patient had a GB stone with acute cholecystitis, a CBD stone, and a history of failed endoscopic treatment caused by altered anatomy. This set of circumstances led us to develop a new innovative 1-step procedure for the concurrent removal of the GB and CBD stones.

EST has the disadvantage of requiring a sphincterotomy which leads to loss of normal papilla sphincter function, with the potential for chronic complications, such as duodeno-biliary reflux, inflammation of the biliary tree, and ampullary stenosis.^[7]^ The use of EST is also limited in patients with a history of failed cannulation of the bile duct, a narrowed upper gastrointestinal tract, or surgically altered anatomy, as the increased length of the afferent and efferent limbs makes it difficult to reach the duodenal papilla.^[4]^ A 19-year review of ERCP in patients with altered gastrointestinal anatomy from a single center reported a successful duodenal intubation rate of 51.9%, with failure resulting from the inability to cannulate (44%) or to identify the papilla (39.6%).^[8]^ Percutaneous transhepatic papillary balloon dilatation is an alternative interventional radiological procedure for these cases. The technique was first reported by Staritz et al^[9]^ in 1983. In our previous research on this procedure,^[10]^ we reported that PTBT was associated with an overall low incidence of complications, which included: biliary tract infection (3/68 of cases), 4.4%; hemorrhage (2/68 of cases), 2.9%; and pancreatitis (1/68 of cases), 1.4% (1/68 of cases). Stones recurred in 10/68 of cases (14.7%), and reflux cholangitis occurred in 4/68 of cases (5.8%). No severe complications occurred, including gastrointestinal or biliary tract perforation or severe pancreatitis, and the technique preserved the function of the sphincter of Oddi.

Since its introduction by Dubois et al^[11]^ in 1989, LC has become the first-choice treatment for cholecystolithiasis, replacing open surgery. Compared to open surgery, LC has the advantage of a shorter postoperative recovery and lower morbidity.^[12]^ However, the use of LC for the treatment of acute cholecystitis is not considered routine, with debate regarding the ideal timing and its advantages over surgical management.^[13]^ The conversion rate for delayed LC in the treatment of acute cholecystitis is higher at 51% than that for early LC at 19%.^[14]^ Concurrent LC and laparoscopic removal is also effective and safe for the removal of CBD stones.^[6]^ However, laparoscopic CBD exploration requires higher technical skill than transcystic stone removal.^[15,16]^

B-type ultrasound-guided percutaneous transcystic intervention was introduced in the early 1980s^[17,18]^ and has since been widely accepted as an effective solution for stone removal in patients with acutely inflamed gallbladders,^[19]^ allowing various interventional manipulations to be performed following successful negotiation of the cystic duct.^[20]^ Gil and Eli have also reported on their successful experience of removing stones in the biliary tree through an existing gallbladder drain or T-tube, reporting a success rate of more than 90%.^[21,22]^ The following key procedural points should be considered to ensure success. First, the patient should be placed in a supine position and the guide wire and catheter placed into the duodenum through the cystic duct, intrahepatic duct, or CBD. Second, to expand the sphincter of Oddi, the balloon should be positioned accurately and fully dilated. In principle, the diameter of the balloon should be equal to or slightly larger than the diameter of the stone. The sphincter should be dilated slowly as to prevent acute tearing of the muscle fibers. Third, in cases with multiple stones, stones should be removed one-by-one to avoid the possibility of pancreatitis caused by stone debris returning to the pancreatic duct. Fourth, a cystic drainage should be maintained *in situ* to reduce biliary pressure and bile leakage as a means of lowering the risk of the development of pancreatitis. Also, a gallbladder drain should be regularly retained, which also decreases biliary pressure, as well as reducing the incidence of bile leakage and pancreatitis. The main limitation of this combined approach is anatomical variations, including a very tortuous cystic duct, small-caliber cystic duct (<2 mm), the presence of Heister valves, and the lack of structural support from surrounding organs, which decrease the success rate of cannulation of the cystic duct.^[23]^ Moreover, the size of the GB and CBD stones must be considered as an important limitation. Based on our experience and other published reports, the combined approach is recommended for GB stones with a diameter <14 mm and CBD stones <22 mm in diameter.^[10,20–22]^

Based on the limited evidence available, PTBD combined with a percutaneous transcystic approach may provide an alternative interventional procedure for concurrent GB and CBD stones, being of specific value for patients with previously altered gastrointestinal anatomy and acute cholecystitis.
